# The change of longitudinal relaxation rate in oxygen enhanced pulmonary MRI depends on age and BMI but not diffusing capacity of carbon monoxide in healthy never-smokers

**DOI:** 10.1371/journal.pone.0177670

**Published:** 2017-05-11

**Authors:** Simon Sven Ivan Kindvall, Sandra Diaz, Jonas Svensson, Per Wollmer, Lars E. Olsson

**Affiliations:** 1 Medical Radiation Physics, Translational Medicine, Lund University, Malmö, Sweden; 2 Medical Radiology, Translational Medicine, Lund University, Malmö, Sweden; 3 Medical Imaging and Physiology, Skane University Hospital, Lund, Sweden; 4 Clinical Physiology, Translational Medicine, Lund University, Malmö, Sweden; Linköping University, SWEDEN

## Abstract

**Objective:**

Oxygen enhanced pulmonary MRI is a promising modality for functional lung studies and has been applied to a wide range of pulmonary conditions. The purpose of this study was to characterize the oxygen enhancement effect in the lungs of healthy, never-smokers, in light of a previously established relationship between oxygen enhancement and diffusing capacity of carbon monoxide in the lung (D_L,CO_) in patients with lung disease.

**Methods:**

In 30 healthy never-smoking volunteers, an inversion recovery with gradient echo read-out (Snapshot-FLASH) was used to quantify the difference in longitudinal relaxation rate, while breathing air and 100% oxygen, ΔR1, at 1.5 Tesla. Measurements were performed under multiple tidal inspiration breath-holds.

**Results:**

In single parameter linear models, ΔR1 exhibit a significant correlation with age (p = 0.003) and BMI (p = 0.0004), but not D_L,CO_ (p = 0.33). Stepwise linear regression of ΔR1 yields an optimized model including an age-BMI interaction term.

**Conclusion:**

In this healthy, never-smoking cohort, age and BMI are both predictors of the change in MRI longitudinal relaxation rate when breathing oxygen. However, D_L,CO_ does not show a significant correlation with the oxygen enhancement. This is possibly because oxygen transfer in the lung is not diffusion limited at rest in healthy individuals. This work stresses the importance of using a physiological model to understand results from oxygen enhanced MRI.

## Introduction

In 2012, chronic obstructive pulmonary disease (COPD) alone was the third largest cause of death world-wide (3.1 million) [1] and the prevalence of COPD is predicted to increase over the next decades due to an ageing population and continued exposure of populations to risk factors such as tobacco smoke and pollutants [2]. Although COPD is a mix of chronic bronchitis and emphysema, which effects ventilation, perfusion and diffusion in a spatially heterogeneous manner, the disease is often classified according to spirometric pulmonary function tests (PFT) based on ventilation [2], with little regard to perfusion and diffusion of gases. In this context, oxygen enhanced (OE) -MRI is a promising modality for imaging pulmonary function [3]. As described in detail by Ohno and Hatabu [4], molecular oxygen will have the effect of a paramagnetic contrast agent in pulmonary blood according to R_1_ = R_1,0_+r_1,O2_∙PO_2,_ where R_1_ = 1/T_1_ is the measured longitudinal relaxation rate; R_1,0_ is the baseline relaxation rate; PO_2_ is the partial pressure, and r_1,O2_ is the relaxivity, of oxygen. The relaxation enhancement (OE-effect), quantified as the change in relaxation rate between breathing air and oxygen, ΔR_1_, is claimed to be proportional to the corresponding change in oxygen partial pressure, ΔPO_2_ [5]. The clinical relevance of OE-MRI has been previously attributed to a high correlation between OE (as signal enhancement) and the relative predicted carbon monoxide diffusing capacity of the lung (RP D_L,CO_) [6,7].

During recent years, OE-MRI has successfully been used to study patients with COPD [8,9]; chronic lung allograft dysfunction [10]; interstitial lung disease [11]; and asthma [12] as well as a murine emphysema model [13]. Several centers have also used ultra-short echo time for OE-measurements, to address the inherently low MR-signal [14–16]. However, although OE-MRI has been applied to study disease and healthy controls [15,17], no previous work has described the effect with respect to age and sex in a healthy cohort. Moreover, the original description OE-effect as a pure relaxation enhancement [4], fails to take into account some physiological considerations:

A description of the OE effect often includes a previously reported (in 2002) dependency of OE on RP D_L,CO_ in two mixed cohorts of patients and volunteers [6,7]_._ However, the transfer of oxygen in the healthy lung is perfusion limited at rest—in contrast to the transfer of carbon monoxide which is diffusion limited [18]–and the arterial PO_2_ (Pa_O2_) has recently (in 2015) been shown to *not* be related to RP D_L,CO_ in healthy individuals at rest [19]. It is important to note that Pa_O2_ is expected to be the same as the pulmonary PO_2,_ apart from the effects of physiological shunt.

As outlined in the Theory section, the OE effect is expected to depend on the volumes of pulmonary arterial and venous blood. Since the measured baseline lung T_1_ has been shown to also depend on lung blood T_1_[20]; lung blood volume changes with age and sex [21]; and lung T_1_ depends on age and sex[22]; the OE-effect in the lung will likely depend on age and sex.

Finally, Pa_O2_ is known to vary with body mass index (BMI) even in non-obese subjects [23], with high-BMI subjects having a lower Pa_O2_ [24]. This potentially makes BMI a predictor of the OE-effect in a healthy cohort, assuming that OE-measurements do indeed reflect the transfer of oxygen to the blood.

The purpose of this paper is to present and describe novel data on oxygen enhanced MRI, quantified by ΔR_1_, in the lungs of healthy never-smokers with respect to age, sex and BMI, and to evaluate the data in view of a previously reported relationship between the OE-effect and RP D_L,CO_ [6,7].

### Theory

The original description of OE-MRI relies on the paramagnetic effect of dissolved oxygen in blood, which would give a signal enhancement proportional to the change in oxygen partial pressure in lung blood [4]. However, this does not take into account the macromolecule dependent relaxivity of oxygen; the presence of deoxygenated (precapillary) blood in the lung; and the changes in blood volume that may come with oxygen breathing.

The relaxivity of molecular oxygen, r_1,O2,_ is often cited to be *2*.*49 x 10*^*−4*^ s^-1^ mmHg^-1^ [8,25]; but this applies to distilled water [5], and the value in whole blood at 1.5 Tesla is likely between 3.38 to 4.38 x *10*^*−4*^ s ^-1^ mmHg^-1,^ depending on the erythrocyte volume fraction (EVF) [5,26,27]. The difference in O_2_-relaxivity between blood and water can be appreciated with the Bloomberg-Purcell-Pound relaxation theory, since para- and diamagnetic relaxation is synergistic [28,29]. Using a relaxivity value from whole blood, 0.41 EVF, r_1,O2_ = 4.1 x *10*^*−4*^ s ^-1^ mmHg^-1^ [27], the ΔR_1_ in pulmonary veins is expected to be approximately 0.2 s^-1^ for a 500 mmHg change in PO_2_ –twice as much as reported in the lung [15]. However, while the ΔPO_2_ in pulmonary veins is theoretically more than 500 mmHg, the ΔPO_2_ in pulmonary arteries is close to 15 mmHg [18]. The blood relaxation in turn is quite well understood and described by a plasma- and erythrocyte-water compartment in fast exchange [30,31]. Thus, the pulmonary arterial compartment (deoxygenated blood) will exhibit a small *decrease* in R_1_ with 100% oxygen breathing due to the oxygenation of hemoglobin [30,31]. Moreover, the overall blood content of the lung seems to increase with oxygen supplementation [32], and since the lung T1 is an echo time dependent mix of the lung parenchyma T1 and blood T1 [20], this mechanism will *decrease* the overall lung R_1_, but increase signal intensity. The observed *increase* in lung R_1_ (global ΔR_1_ on the order of 0.10 s^-1^) is explained by the large signal contribution of oxygenated blood within most voxels of the lung.

Considering this, the OE-effect does depend on lung P_O2_; but one must consider the total blood content and partitioning between the oxygenated/deoxygenated (pulmonary arterial/venous) blood compartments of the lung voxel where the T1-calculation is done.

## Methods and materials

### Subjects and hardware

With approval from the Regional Ethical Review Board, 31 never-smoking healthy subjects (16 male, 15 female) between 20–70 years were recruited to perform a clinical lung MRI examination, pulmonary function tests (PFT) and whole lung T_1_ measurements with and without oxygen enhancement. Never-smokers were defined according to three criteria: a) never smoked daily for more than a month, b) smoking occasionally less than once a month, and c) reporting 0 pack-years of lifetime tobacco use. Subjects were provided with written instructions, signed informed consent, reported lifetime tobacco use, pulmonary health status and filled out a MRI safety sheet prior to the visit. The PFT was either made immediately after the MRI examination, or the next day. All MRI measurements were made on a 1.5 Tesla Siemens Magnetom AvantoFit (SIEMENS Healthcare, Erlangen, Germany), with an 18 channel body coil and a 32 channel spine matrix.

### Clinical examination and PFT

All subjects underwent a morphological MRI examination, which included two full coverage coronal HASTE (Half-Fourier Acquisition Single shot Turbo Spin Echo) acquisitions during end-inspiration breath-hold and during free breathing respectively, as well as an axial VIBE acquisition during end-inspiration breath-hold. The images were examined by clinical radiologists with 10 or 5 years of experience. Pulmonary function tests including measurements of total lung capacity (TLC), residual volume (RV), functional residual capacity (FRC), vital capacity (VC), forced expiratory volume in 1 second (FEV_1_), D_L,CO,_ and D_L,CO_ adjusted for alveolar volume (k_CO_), were performed by clinical physiology personnel, using a Carefusion Masterscreen PFT and Body/Diffusion and the SentrySuite version 2.7 or higher (Carefusion Corporation, San Diego, CA). Relative predicted D_L,CO_ was calculated using standard equations from the European community for steel and coal (ECSC) [33], other values are reported as nominal volumes.

### T_1_-measurements and oxygen enhanced MRI

All T_1_ measurements were made with the Snapshot FLASH pulse sequence[34] which is based on the Look and Locker sequence [35]. Following spin inversion, several gradient echo images are collected during magnetization recovery. Imaging parameters were as follows: matrix 128 x 64 zero filled to 256 x 256; 450 mm square field of view; 1.5 cm slice thickness; TE = 0.67 ms; TR = 3.0 ms; and flip angle = 7°. A non-selective hyperbolic secant pulse was used for spin inversion and Hanning-windowed sinc pulses with 1.6 sidelobes were used for image acquisition. One T_1_ measurement is made in each slice; 16 gradient echo images with different inversion times are used in the calculation of the T_1_ map; and the total measurement time is 3.0 seconds per slice. Each T_1_ measurement was preceded by oral instructions for breath-hold after a tidal inspiration and subjects were given at least 10 seconds of free breathing in between measurements, which is also assumed to be enough to restore magnetization equilibrium for T_1_ < 2000 ms.

T_1_ mapping of the entire lung was performed at baseline and after 5 min inhalation of 100% oxygen. Medical air or oxygen was supplied at >10 l/min using a tight fitting oro-nasal mask with a non-rebreathing valve (V2-Mask 7450, T-Shape valve 2700, Hans Rudolph Co., Kansas City, MO), equipped with a 1 liter plastic reservoir bag and 40 cm low-resistance tubing (dead space in valve housing is 77 ml). Three sizes of masks were available to ensure optimal fit to each subject; each subject tried to inhale while shutting the air-inlet with their hand and reported no leakage prior to measurement.

To estimate the uncertainty of the measurements, a single slice is imaged repeatedly 10 times, using a rapid dynamic acquisition protocol with breath-hold [36].

### Data processing

Data processing was made in MatLab R2014b (MathWorks, Natick, MA). The lungs were segmented in 3D using a region growing algorithm (“regionGrowing”, Daniel Keller, 2011) or, in case the first algorithm fails, a slice by slice k-means clustering algorithm with five clusters. Either segmentation method was applied on a 3D image stack, containing the last magnitude image from the series of collected gradient echoes, followed by manual removal of major vessels. Segmentations were considered successful when each slice clearly included the signal magnitude gradient representing the border of the lung. The T_1_-value of the entire lung volume is defined as the center of a Gaussian curve, fitted to the histogram of all voxel values in the lung segmentation after a histogram thresholding [17,22]. Using the T_1_-values at baseline and after 5 min oxygen inhalation, the global difference in relaxation rate, ΔR_1_, was calculated for each subject.

### Correcting for lung inflation

Using the segmented lung from the 3D-stack of magnitude images, the total lung volume at the time of measurement can be calculated. Since it is known that global T_1_ varies with lung inflation [17], the difference in lung volume between breathing oxygen and air, **ΔV**_**L**_ **= V(oxygen)−V(air)**, was calculated for each subject.

### Statistical evaluation

Linear models of ΔR_1_, and ΔR_1_ adjusted for age and sex, as a function of PFT-parameters were tested for 24 subjects with complete data. Linear models of general demographics: age, sex, height, weight and BMI, as well as ΔV_L_ were tested for the whole group of 30 subjects.

A stepwise linear regression is applied to a model of age, sex, BMI and ΔV_L_−including first order interaction terms. Interaction terms are removed one by one based on the ANOVA p-value (likelihood that the estimate is separate from zero) to maximize the adjusted coefficient of determination (adj. R^2^). The linear model ΔR_1_ as a function of RP D_L,CO_ is also tested for sex confounding and presented.

No correction is made for multiple testing and nominal p-values are used consequently.

### Calculation of error bars

Using the rapid acquisition protocol [36] during steady state, 9 images of a single central slice are collected and the coefficient of variation (CV) of the mean R_1_ in a circular ROI in the top right lung is calculated. The error bars represent ± CV x R_1_(air) for each subject.

## Results

All subjects completed the MRI examinations; six subjects were not able to complete the PFT due to logistical reasons; one subject was completely excluded due to clinical findings. General demographics and PFT measurements of the cohort has been presented earlier [22].

**Fig 1:**
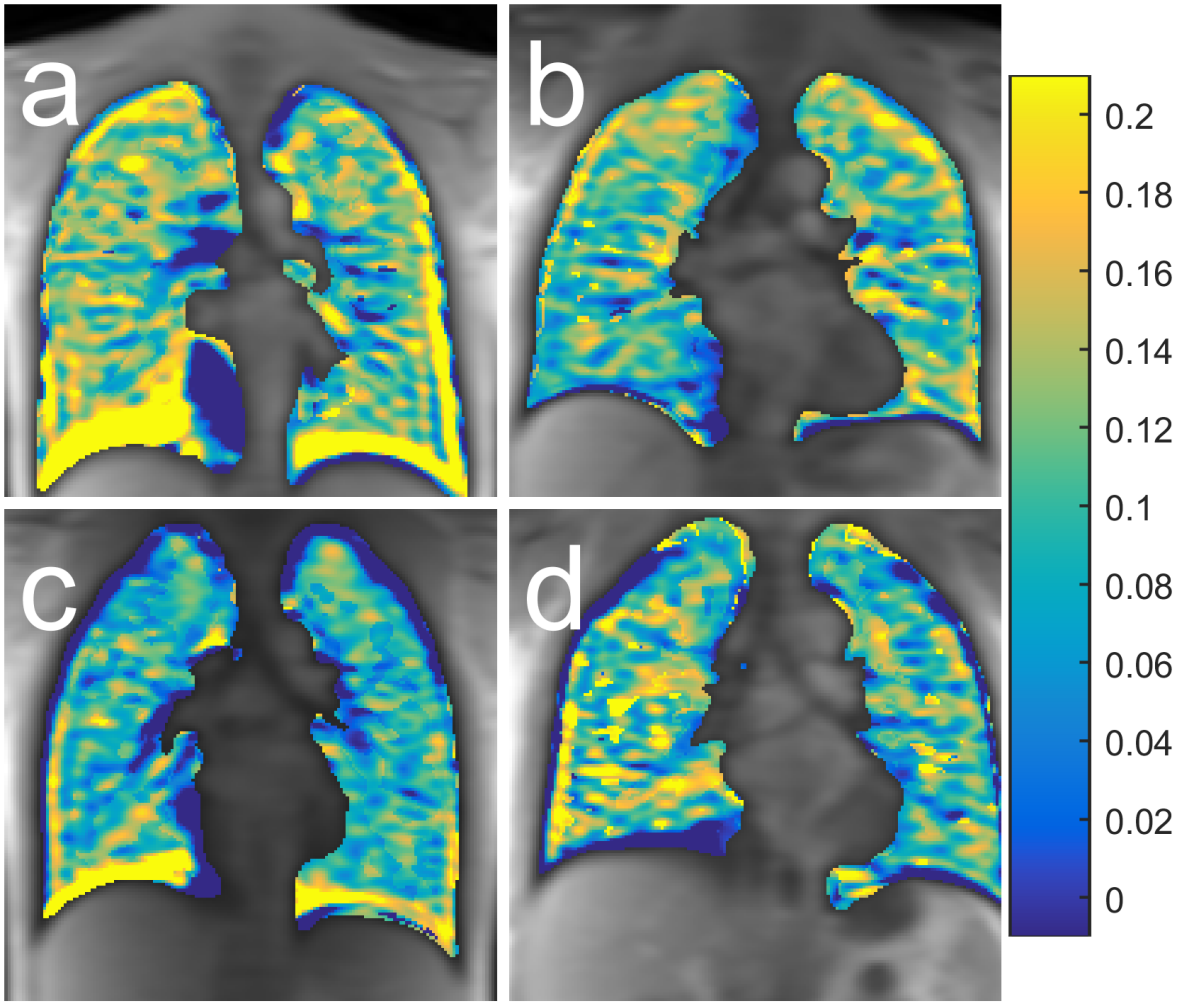
Oxygen enhancement images from four subjects. Representative maps of ΔR_1_ = R1,oxygen−R_1,air_ for a) Female age <30 years, b) Female age >45 years, c) Male age <30 years and d) Male age >45 years. The images are provided for completeness, and are not registered or processed.

Plain unregistered maps of ΔR_1_ = R1,oxygen—R_1,air_ are presented in Fig 1 for representative females and males of age <30 and age >45. It is interesting to note that the pulmonary trunk and arteries exhibit negative enhancement (R_1,oxygen_ < R_1,air_) in most subjects. The hypo/hyperintense regions close to the diaphragm result from misalignment between oxygen and air-images which is not corrected for at this stage, since difference images are not used in any calculations. The R^2^ and p-values of single parameter linear models, and age- and sex-adjusted models, of nominal PFT parameters are presented in Table 1, for 24 subjects with complete PFT.

**Table 1:** Values of R^2^ for ΔR_1_ as a function of pulmonary function test results for n = 24 subjects with complete pulmonary function tests. Unadjusted linear models, and models adjusted for age and sex are presented.

	Unadjusted:		Age and sex adj.:	
	R^2^	P	R^2^	P
**Age+sex**	0.35	0.011	---	---
**Sex**	0.032	0.407	---	---
**Age**	0.323	0.004	---	---
**BMI**	0.449	3.4e-3	0.599	3.2e-3
**TLC**	0.074	0.197	0.355	0.030
**RV**	0.291	0.007	0.365	0.026
**FRC**	0.005	0.742	0.392	0.017
**VC**	0.009	0.665	0.352	0.031
**FEV**_**1**_	0.023	0.477	0.351	0.031
**D**_**LCO**_	0.003	0.805	0.352	0.031
**RP D**_**LCO**_	0.043	0.333	0.349	0.032
**K**_**CO**_	0.026	0.454	0.359	0.028

Abbreviations: body mass index, total lung capacity, residual volume, functional residual capacity, vital capacity, forced expiratory volume in 1 second, diffusing capacity of the lung for carbon monoxide (D_L,CO_), relative predicted D_L,CO_ and D_L,CO_ corrected for alveolar volume

The mean ΔR_1_ for 30 subjects is 0.11 s^-1^ with SD 0.021 s^-1^. Single parameter linear models of age, sex, height, weight, BMI and ΔV_L_ are presented in Table 2, for all 30 subjects. The linear model ΔR_1_ versus age yields a statistically significant slope of -0.00076 s^-1^ year^-1^ (95% CI [-0.0012–0.00029] s^-1^ year^-1^, p = 0.003, n = 30) corresponding to a 6.9% decrease in ΔR_1_ in10 years. The linear model ΔR_1_ versus BMI yields a statistically significant slope of -0.00431 s^-1^ (kg m^-2^)^-1^ (95% CI [-0.0065–0.0021] s^-1^ (kg m^-2^)^-1^, p = 0.0004, n = 30) corresponding to 3.9% decrease in ΔR_1_ for one BMI unit. Sex alone is not a significant predictor for ΔR_1_ in the whole group (T-test, p = 0.43).

**Table 2:** Values of R^2^ for linear model of ΔR_1_ versus general demographics, for n = 30 subjects.

	R^2^	P-value
**Sex**	0.022	0.43
**Age**	0.28	0.0027
**Height**	0.008	0.63
**Weight**	0.28	0.0027
**BMI**	0.37	0.0004
**ΔV**_**L**_	0.17	0.02

**Fig 2:**
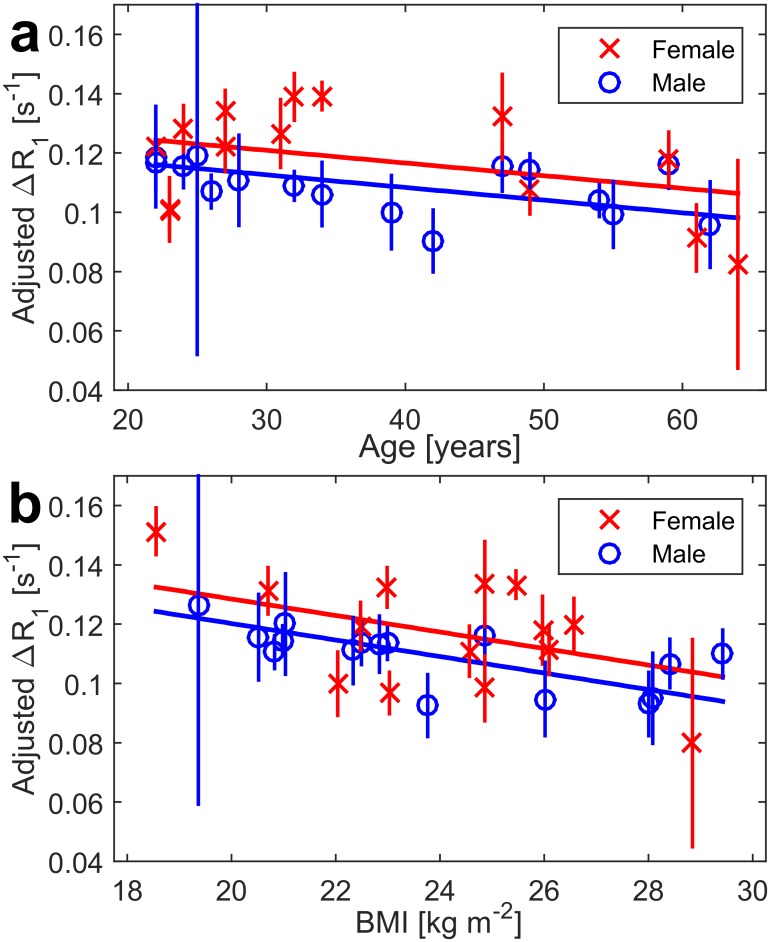
Main result plots of OE as a function of age and BMI. Equation: *ΔR*_*1*_ *= ΔR*_*1*,*0*_*(sex) + C*_*1*_*Age + C*_*2*_*BMI + C*_*3*_*Age x BMI*+ C_4_ΔV_L_, whole model R^2^ is 0.66 at p = 5e-5 for n = 30 participants. Error bars represent the coefficient of variation in repeated measurements of R_1_ a) ΔR_1_ adjusted for BMI and **ΔV**_**L**_ as function of age and sex *b)* ΔR_1_ adjusted for age and **ΔV**_**L**_ displayed as function of BMI.

The final model contains sex, age, BMI, ΔV_L_, and the BMI x age interaction term, where the removal of further terms will decrease the adjusted R^2^. The model is described by ΔR_1_ = ΔR_1_(male/female) + C_1_Age + C_2_BMI + C_3_Age x BMI + C_4_ΔV_L_; has R2 = 0.66 at p = 5e-5; and is presented in Fig 2, with model estimates in Table 3. It is important to note that ΔR_1_ *decreases* with age and BMI, despite the C_1-2_ values being positive, and that the age-, BMI- and interaction-coefficients are not statistically different between sexes. It is also important to note that neither sex nor ΔV_L_ have an effect at p<0.05, but including them raises the adjusted **R**^**2**^ of the whole model. The mean CV of all subjects is 1.4%, n = 30. Since the CV for each subject is not correlated with any of the variables age, BMI, sex, ΔV_L_ or ΔR_1_ (p>0.3), it is not incorporated in the statistical analysis, but only provided as a visual aid for interpretation.

**Table 3:** Model parameters of ΔR_1_ = ΔR_1_(sex) + C_1_Age + C_2_BMI + C_3_Age x BMI + C_4_ΔV_L_ for n = 30, with 95% confidence intervals.

	Estimate	95% CI	p-value
**ΔR**_**1,0**_**(male) [s**^**-1**^**]**	0.0256	[-0.10 0.16]	0.69
**ΔR**_**1,0**_ **(female)[s**^**-1**^**]**	0.0339	[-0.09 0.16]	0.59
**C**_**1**_ **[s**^**-1**^ **y**^**-1**^**] x 10**^**−3**^	3.97	[0.8 7.1]	0.017
**C**_**2**_ **[s**^**-1**^ **kg**^**-1**^ **m**^**2**^**] x 10**^**−3**^	4.20	[-1.3 9.7]	0.13
**C**_**3**_ **[s**^**-1**^ **kg**^**-1**^ **m**^**2**^ **y**^**-1**^**] x 10**^**−3**^	-0.183	[-0.31–0.05]	0.008
**C**_**4**_ **[s**^**-1**^ **l**^**-1**^**] x 10**^**−3**^	10.8	[-7.3 29]	0.23

**Fig 3:**
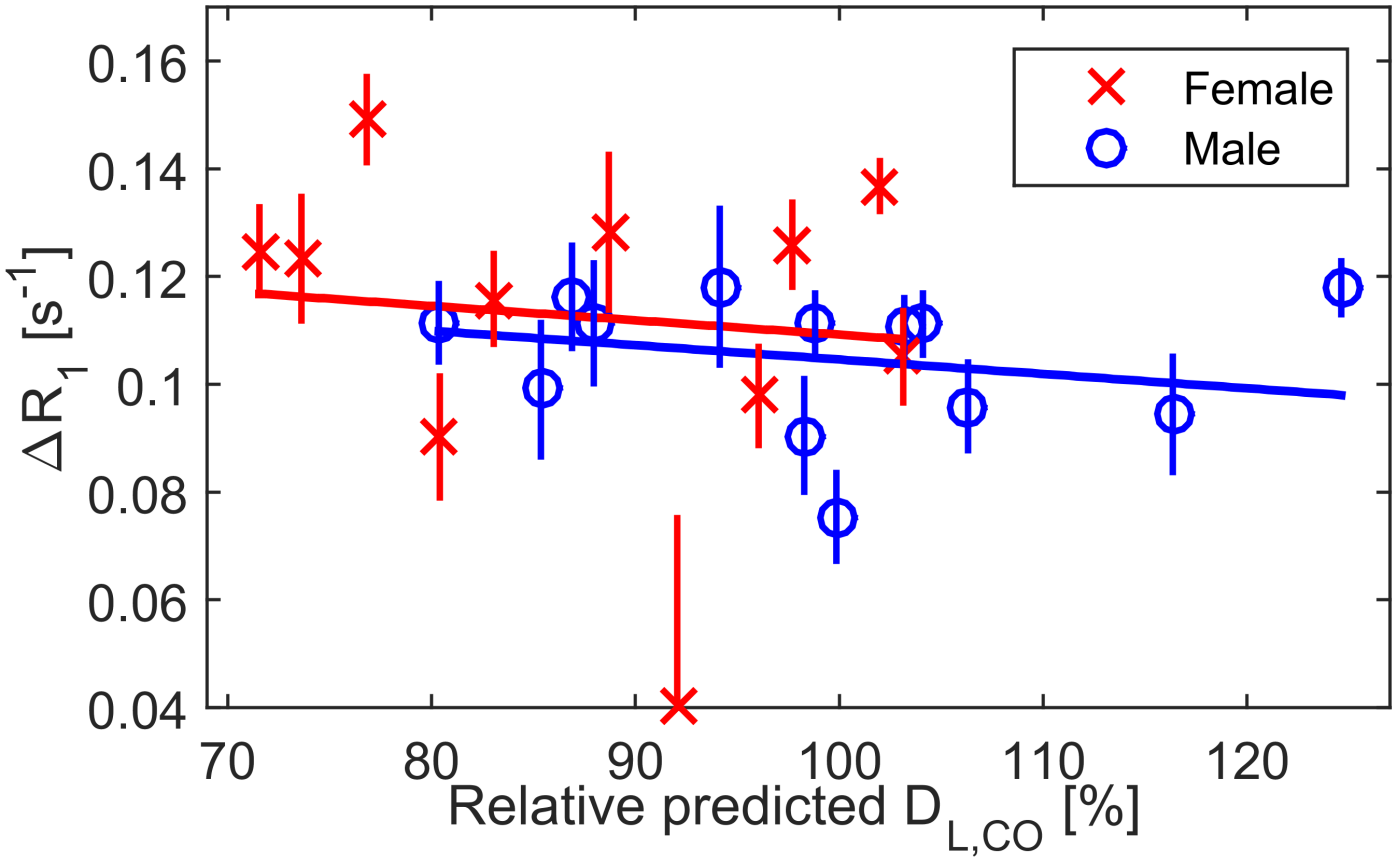
Plot of OE as a function of diffusing capacity. Linear regression of ΔR_1_ as function of relative predicted D_L,CO_ and sex for n = 24 participants, whole model R^2^ is 0.05 at p = 0.57. Neither sex nor D_L,CO_ is a significant predictor in this model. Error bars represent the coefficient of variation in repeated measurements of R_1_.

A linear model of ΔR_1_ as a function of RP D_L,CO_ is not significant; p = 0.33, with slope -0.00035 s^-1^ [%]^-1^ (95% CI [-0.0011 0.00037] s^-1^ [%]^-1^). When allowing different baseline ΔR_1_ based on sex, the effect of RP D_L,CO_ on ΔR_1_ is even smaller, with slope -0.00027 s^-1^ [%]^-1^ (95% CI [-0.0011 0.00055] s^-1^ [%]^-1^) at p = 0.51, and whole model R^2^ = 0.05, Fig 3.

## Discussion

In this study it has been shown that the oxygen enhancement as quantified by ΔR_1_ decreases with age in a healthy population, with a more prominent decrease in subjects with high BMI. Moreover, ΔR_1_ is not correlated to the relative predicted or nominal D_L,CO_ (single breath, inert gas measurement), in our group of healthy never-smokers, which is congruent with current knowledge of oxygen diffusion in the lung [19].

The mean OE effect in the present study is similar to previous reported values of 0.08–0. 12 s^-1^ [15], and the linear model used to describe the OE-effect seems feasible with respect to the physiological variations in the pulmonary circulation with age, BMI and sex [21,23].

### Oxygen enhancement and D_L,CO_

The lack of correlation between D_L,CO_ and ΔR_1_ in the healthy cohort is surprising at first; but in fact, Pa_02_ is not correlated to RP D_L,CO_ at rest in healthy lungs [19], and a strong relationship between D_L,CO_ and OE is only expected in very dysfunctional lungs or during exercise when oxygen transfer is diffusion limited [18]. A similar case is found in a recent article about T1 as a function of k_CO_ (D_L,CO_ adjusted for alveolar volume) in healthy volunteers and COPD patients [37]; although the overall T1 vs. k_CO_ relationship is strong, the correlation is visibly close to zero for the healthy group alone [37]. Both cases are examples of Simpson’s paradox [38], where a between-group regression is not necessarily valid within a group. It is possible that pathologic tissue change such as emphysema or fibrosis is a common cause for changes in T1, OE and D_L,CO_ in diseased lungs, but that other physiological considerations are more important for the T1, OE and D_L,CO_ in healthy lungs.

### Oxygen enhancement and BMI and age

The arterial oxygen partial pressure will decrease with age and BMI [23], which logically will have a direct effect on the observed OE effect. A common hypothetical explanation for both the age and BMI dependency the OE effect is the closure of small airways, which produces micro-shunting [18]. The ageing lung loses elastic recoil (increased compliance) which aggravates this effect [18], and visceral fat may further compress the lung in the supine position and produce the age interaction. Shunting can in this context result in an increase in the deoxygenated fraction of lung blood of the given voxel, and in a decrease of the pulmonary venous blood P_O2_ –creating a concurrent effect to lower the OE-effect.

The correlation between BMI and OE may also be a confounding effect of general physical fitness on both pulmonary function and BMI: a sedentary lifestyle or low physical activity increase BMI in overweight (25<BMI<30) individuals [39], and there is a positive dose-response relationship between physical activity and lung function as quantified by FEV_1_ [40].

### Limitations

A major limitation of the study is sample size, and the slightly skewed age distribution. However, since we required subjects to fill out a questionnaire about smoking, as outlined in the methods section, very few prospective volunteers born before the 70’s identified themselves as never-smokers. The lung function as quantified by PFT is normal for all volunteers; residual volume increases with age (ρ = 0.7 at p<0.01); and k_CO_ decreases with age (ρ = -0.4 at p = 0.015) [22].The error bars represent the CV of the T1-measurement, which mostly depend on the subject’s breathing technique. Respiratory phase can alter the T1-value by at least 6% [17], but the mean CV of the cohort presented here was lower than 2%, indicating that subsequent breath-holds resulted in a very similar lung volume. However, we did choose to correct for differences in lung inflation between oxygen and air measurements, which were separated by 5 minutes of free breathing to ensure gas equilibrium.

Previous work have only compared OE and the RP D_L,CO_ [6,7], however, in the healthy cohort of the present study, none of the parameters RP D_L,CO_, D_L,CO_ or k_CO_ are significantly correlated with the measured OE-effect. As seen in Table 1, age and sex are much more potent predictors alone, and adding RP D_L,CO_, D_L,CO_ or k_CO_ has no impact on the model R^2^.

The OE measurement in this study was performed during a tidal inspiration breath-hold (FRC + tidal volume), and, as the magnitude of the OE effect likely depends on the respiratory phase [15], any work on the OE-effect during other parts of the respiratory cycle should take this into account. In this study, the difference in total volume of the segmented lung at air or oxygen measurement was used as a model parameter.

Finally, this entire paper has been regarding a *global* measure of the OE effect in the lungs of healthy individuals. When studying patients it is advisable to employ a method that allows the detection of regional heterogeneity, as this is expected in disease [8,10,13]. In this study, coronal T1-maps were collected since this method allows the whole lung to be sampled in the shortest time. Ideally, OE-MRI should be performed with full lung coverage, as we have done, but with isotropic resolution to account for gravity dependent heterogeneity—which is expected even in the supine healthy lung [18].

### Conclusion

Further studies should aim to improve the regional quantification possibilities of OE-MRI and describe the underlying physiological processes responsible for the changes in T1, OE and pulmonary function (such as D_L,CO_) in the healthy and diseased lung. Eventually, a hemodynamic model of the lung is necessary to interpret all OE-MRI data—using either relaxation- or signal enhancement as end-points.

In conclusion, we have shown that OE-MRI as quantified by ΔR_1_ varies with age and BMI, but not with relative predicted D_L,CO_, in a healthy, never-smoking group. Moreover, a model including sex, age, BMI, lung volume and an age-BMI interaction is optimal, with respect to the adjusted R^2^-value, to describe the OE effect in this cohort. Thus, the influence of these variables should be considered in OE-studies of healthy volunteers or patients. The overall interpretation of the present study is that closure of small airways is more important for the OE-effect than gas diffusion, but more sophisticated experiments are needed to confirm this hypothesis.

## Supporting information

S1 File Supplementary raw data file. Anonymous data from all subjects are included for availability at the Journal’s webpage.
(TXT)
